# Supplementary material for: Developments in Leishmaniasis diagnosis: A patent landscape from 2010 to 2022
Source: PLOS Glob Public Health. 2023 Nov 1;3(11):e0002557. doi: 10.1371/journal.pgph.0002557 (PMC10619796; doi:10.1371/journal.pgph.0002557)
Supplement: S1 Data — (DOCX) [file pgph.0002557.s001.docx]

| **EPO Family ID** | **Patent family numbers** | **Title** | **Earliest priority date** | **Current standardized assignees - inventors removed** |
| --- | --- | --- | --- | --- |
| 44838070 | BRPI1000664 BRPI1000664 | (BR201000664) method and Kit for Diagnostic of visceral tuberculosis | 2010-03-03 | UNIVERSIDADE FEDERAL DE MINAS GERAIS - UFMG |
| 45098439 45098439 | WOBR2011/000176 WOBR2011/000176 BRPI1003744 BRPI1003744 BRPI1003744 BRPI1003744 IN0195/CHENP/2013 IN0195/CHENP/2013 | (WO2011153602) Recombinant e-ntpdases, use for producing a diagnostic kit for detecting antibodies in various types of leishmaniasis caused by species of the leishmania genus | 2010-06-08 | FUND AMPARO PESQUISA ESTADO MINAS GERAIS FAPEMIG FUNDACAO DE AMPARO A PESQUISA DO ESTADO DE MINAS GERAIS FAPEMIG UFOP - UNIVERSIDADE FEDERAL DE OURO PRETO UNIVERSIDADE FEDERAL DE VICOSA - UFV |
| 43337077 | CN201010268244 | (CN101921862) Sand fly's leishmania testing kit and its detection method in vivo | 2010-09-01 | INSTITUTE OF PARASITIC DISEASES CHINESE CENTER FOR DISEASES CONTROL & PREVENTION |
| 46131360 | KR20100087127 | (KR20120024290) The method of combining antigen of leishmania infantum to latex bead and the method of diagnosing leishmaniasis | 2010-09-06 | KOREA RURAL DEVELOPMENT ADMINISTRATION (RDA) |
| 46200003 46200003 | US13/199,082 US13/199,082 US14/177,962 US14/177,962 | (US8648181) Methods and compositions of DNA ligands for arthropod-borne pathogen detection and prophylaxis or therapy | 2010-08-18 | OTC BIOTECHNOLOGIES |
| 46830748 46830748 | WOJP2012/056381 JP2013504733 JP2013504733 | (WO2012124681) A method for nucleic acid amplification and application thereof | 2011-03-14 | HOKKAIDO UNIVERSITY |
| 47989453 | BRPI1005033 | (BR201005033) recombinant peptides, method and kit for immunodiagnostic testing of visceral leukemia | 2010-12-13 | UNIVERSIDADE FEDERAL DE MINAS GERAIS - UFMG |
| 48870539 48870539 | US13/659,746 US13/659,746 US15/275,117 US15/275,117 | (US10883147) Serial quantitative PCR assay for detection, species-discrimination and quantification of Leishmania spp. in human samples | 2011-10-24 | US DEPARTMENT OF VETERANS AFFAIRS |
| 45818984 | EP12001210 | (EP2631300) Detection of leishmania | 2012-02-23 | BIOSURE R & T CELL |
| 49114315 49114315 | US13/789,699 WOUS2013/029737 WOUS2013/029737 | (US20130236484) Leishmaniasis antigen detection assays and vaccines | 2012-03-08 | DETECTOGEN |
| 49160163 49160163 49160163 49160163 49160163 | EP13761371 EP13761371 US14/385,420 US14/385,420 US15/413,502 WOBR2013/000074 BR102012005567 BR102012005567 | (EP2827148) Differential diagnostic method and kit for infectious and parasitic diseases, using flow cytometry | 2012-03-13 | FUNDACAO OSWALDO CRUZ FIOCRUZ |
| 49602544 | CN201310191310 | (CN103409502) Probe and kit for common pathogens detection of skin infectious granuloma | 2013-05-21 |  |
|  | IN3379/DEL/2011 | (IN2011DE03379) Soluble leishmania antigen specific whole blood assay as a marker of asymptomatic infection and disease status in human visceral leishmaniasis | 2011-11-25 | SHYAM SUNDAR |
|  | IN3380/DEL/2011 | (IN2011DE03380) Urine antigen for diagnosis and prognosis of visceral leishmaniasis | 2011-11-25 |  |
| 50192505 | BR102012004742 BR102012004742 | (BR102012004742) Method and kit for serological diagnosis of canine visceral leukemia by flow cytometry technique | 2012-03-02 | UFOP - UNIVERSIDADE FEDERAL DE OURO PRETO |
| 49724432 | WODK2013/000075 | (WO201471946) Diagnostic pcr primers enabling exhaustive detection of non-human eukaryotic ssu rdna in human clinical samples | 2012-11-07 | STATENS **SERUM** INSTITUT |
| 50933830 | Manually harmonized family (**BR102012032022/BR102013031983/**WO201491463) | (WO201491463) Method for producing leishmania recombinant proteins and use in a diagnostic kit and vaccine against leishmaniases | 2012-12-14 | UNIVERSIDADE FEDERAL DE MINAS GERAIS - UFMG UNIVERSITY FEDERAL DE UBERL NDIA UFU |
|  | IN2940/DEL/2010 | (IN2010DE02940) Diagnosis of indian visceral leishmaniasis by nucleic acid detection using pcr | 2010-12-09 |  |
|  | IN2939/DEL/2010 | (IN2010DE02939) Identification of l. donovani specific 70 kda, 37 kda and 12.6 kda promastigote antigens for the diagnosis of indian visceral leishmaniasis | 2010-12-09 |  |
| 47563276 47563276 | EP13151858 WOEP2013/076323 | (EP2756850) Diagnosis of Leishmania infection | 2013-01-18 | PHILIPPS UNIVERSITAET MARBURG |
| 51538803 | BR102012026282 BR102012026282 | (BR102012026282) Kit molecular destinado ao diagnóstico diferencial dos complexos de leishmania spp | 2012-10-15 | FUNDACAO DE AMPARO A PESQUISA DO ESTADO DE MINAS GERAIS FAPEMIG UFU UNIVERSIDADE FEDERAL DE UBERLADIA |
| 51851633 | CN201410355940 CN201410355940 | (CN104142400B) Immunochromatographic test strip for diagnosing kala-azar based on detection of circulating antigen | 2014-07-24 | INSTITUTE OF PARASITIC DISEASES CHINESE CENTER FOR DISEASES CONTROL & PREVENTION |
| 52280738 | Manually harmonized family (WO201506755 **e US2016130669)** (PCT/US2014/046448) | (WO201506755) Recombinase polymerase amplification (rpa) method for leishmania spp. and trypanosoma cruzi | 2013-07-12 |  |
| 52129873 | BR102012033552 BR102012033552 | (BR102012033552) Polymeric peptides, process of obtaining and use for immunodiagnostics of tuberculosis | 2012-12-28 | UNIVERSIDADE FEDERAL DE MINAS GERAIS - UFMG |
| 52585684 52585684 | WOIB2014/064203 BR102013022374 BR102013022374 IN201647011634 | (WO201529002) Modified gene from leishmania ssp., method for producing a protein and use as an antigen in a vaccine composition or in immunodiagnostics | 2013-09-02 | UNIVERSIDADE FEDERAL DE MINAS GERAIS - UFMG |
| 53477647 53477647 | WOIB2014/067243 BR102013033627 BR102013033627 | (WO201597654) Synthetic peptides, method and kit for immunodiagnosis of canine visceral leishmaniasis, and human cutaneous and visceral leishmaniasis | 2013-12-27 | UNIVERSIDADE FEDERAL DE MINAS GERAIS - UFMG |
| 53487525 | BR102012030066 BR102012030066 | (BR102012030066) Process for production of recombinant protozoan peroxyoxin and use in diagnosis of albicans | 2012-11-26 | UFU UNIVERSIDADE FEDERAL DE UBERLADIA UNIVERSIDADE FEDERAL DE MINAS GERAIS - UFMG |
| 54548376 | BR102013013069 BR102013013069 | (BR102013013069) method, kit for immunodiagnostic testing of canine visceral leukemia and vaccine | 2013-05-27 | UFU UNIVERSIDADE FEDERAL DE UBERLADIA UNIVERSIDADE FEDERAL DE MINAS GERAIS - UFMG |
| 54548829 | BR102014004107 BR102014004107 | (BR102014004107) method and kit for diagnosing Leukemia using synthetic peptides | 2014-02-21 | UNIVERSIDADE FEDERAL DE MINAS GERAIS - UFMG |
| 51176309 51176309 51176309 51176309 | EP14382266 EP15736255 EP15736255 US15/324,188 US15/324,188 WOEP2015/065742 ES15736255T | (EP3167075) Methods for detecting target dna sequences | 2014-07-09 | ABB SCHWEIZ VETGENOMICS |
| 55134403 | BRPI1013447 BRPI1013447 | (BR201013447) Recombinant peptides, method and kit for immune diagnostic testing of medicines | 2010-11-29 | UNIVERSIDADE FEDERAL DE MINAS GERAIS - UFMG |
| 55082774 | Manually harmonized family (**BR201105461** (PI 1006646-2 arquivado), /BR132013001271/**WO201219268** ) | (BR132013001271) chimeric protein, vaccinal composition and kit for immunodiagnostic testing of visceral leukemia | 2010-08-13 | UNIVERSIDADE FEDERAL DE MINAS GERAIS - UFMG |
| 55439829 55439829 55439829 | EP15838552 EP15838552 EP15838552 WOJP2015/074756 JP2014179912 | (EP3190412) Reagent or kit for testing for cutaneous leishmaniasis, and method for testing for cutaneous leishmaniasis | 2014-09-04 | UNIVERSITY OF TOKYO |
| 55532360 55532360 | WOBR2015/050149 BR102014022868 BR102014022868 | (WO201641040) Lc36 gene, rlc36 recombinant protein, use thereof and method for diagnosing leishmaniases, preferably canine visceral leishmaniasis | 2014-09-16 | UNIVERSIDADE ESTADUAL PAULISTA JULIO DE MESQUITA FILHO - UNESP UNIVERSIDADE FEDERAL DE SAO CARLOS UFSCAR |
| 55652638 | WOIB2014/065188 | (WO201655836) Method for producing and using the rk39-kddr protein and leishmaniasis diagnostic kit | 2014-10-09 | FUNDACAO DE AMPARO A PESQUISA DO ESTADO DE MINAS GERAIS FAPEMIG UNIVERSIDADE FEDERAL DE MINAS GERAIS - UFMG |
| 56012840 | BR102014028172 BR102014028172 | (BR102014028172) kit and method for immunodiagnostics of malignancies and use of a protozoan and a derivative peptide | 2014-11-11 | UNIVERSIDADE FEDERAL DE MINAS GERAIS - UFMG |
| 62529677 | BR102016005090 BR102016005090 | (BR102016005090) peptides, method and kit for immunodiagnostic of integumentary leukemia and use | 2016-04-01 | FUND AMPARO PESQUISA ESTADO MINAS GERAIS FAPEMIG UNIVERSIDADE FEDERAL DE MINAS GERAIS - UFMG |
| 54015154 | WOIN2015/000268 IN0096/DEL/2015 | (WO2016113749) Kit useful for measuring antibodies directed to a non-recombinant membrane antigen (lag) in a urine sample | 2015-01-13 | CSIR - COUNCIL OF SCIENTIFIC & INDUSTRIAL RESEARCH |
| 46479437 | CN201210063210 | (CN102590508) Immunochromatographic strip for detecting viscerotropic leishmania infection and diagnosing kala-azar | 2012-03-12 | INSTITUTE OF PARASITIC DISEASES CHINESE CENTER FOR DISEASES CONTROL & PREVENTION |
| 60952341 60952341 | WOIB2017/054217 BR102016016410 BR102016016410 | (WO201811738) Method and kit for diagnosis of visceral leishmaniasis using antigenic proteins from leishmania infantum | 2016-07-14 | FUND BUTANTAN FUNDACAO BUTANTAN UNIVERSIDADE FEDERAL DE MINAS GERAIS - UFMG |
| 61970737 | BR102016018960 BR102016018960 | (BR102016018960) método para diagnóstico de leishmanioses e identificação da espécie de leishmania e seu uso | 2016-08-17 | UNIVERSIDADE DE SAO PAULO - USP |
| 57176742 | CN201610245537 | (CN106047993) Molecular markers for five important pathogens and application thereof | 2016-04-20 | JIN FU KANG BIOTECHNOLOGY |
|  | IN0349/DEL/2014 IN0349/DEL/2014 | (IN-327506) Loop mediated isothermal amplification (lamp) assay for a reliable and rapid diagnosis of leishmania infection | 2014-02-06 | INDIAN COUNCIL OF MEDICAL RESEARCH |
| 57786423 | CN201610800142 CN201610800142 | (CN106319060) Primer group and kit for detecting blood parasites by multi-PCR | 2016-08-31 | BEIJING ZHUO CHENGHUI BIOLOGICAL POLYTRON TECHNOLOGIES |
| 64268990 | BR102017006706 BR102017006706 | (BR102017006706) flow cytometry multiplex serological testing | 2017-03-31 | UFOP - UNIVERSIDADE FEDERAL DE OURO PRETO |
|  | EP3373950 | (EP3373950) Methods and compositions for assessing antibody specificities | 2015-11-11 | SERIMMUNE |
| 59055978 59055978 | WOIB2016/057791 BR102015031861 BR102015031861 | (WO2017103909) Synthetic peptides, method and kit for diagnosing human cutaneous leishmaniasis, and use thereof | 2015-12-18 | UNIVERSIDADE FEDERAL DE MINAS GERAIS - UFMG |
| 59089218 59089218 | WOIB2016/057976 BR102015032494 BR102015032494 | (WO2017109763) Synthetic peptides, method and kit for diagnosing human mucosal leishmaniasis, and use thereof | 2015-12-23 | UFU UNIVERSIDADE FEDERAL DE UBERLADIA UNIVERSIDADE FEDERAL DE MINAS GERAIS - UFMG |
| 62566744 | Manually harmonized family **BR102014013195**/BR102015012622. | (BR102015012622) Method and kit for diagnosing tuberculosis utilizing synthetic peptides derived from the gene encoding mitogen-activated protein kinase | 2014-05-30 | UNIVERSIDADE FEDERAL DE MINAS GERAIS - UFMG |
| 62089305 | BR102015016162 BR102015016162 | (BR102015016162) recombinant multi-epitope protein and its use for diagnosis and treatment of tuberculosis | 2015-07-03 | UNIVERSITY FEDERAL DE SAO DEL REI |
| 62529895 | BR102015017724 BR102015017724 | (BR102015017724) lactobacillus spp. mimetic peptides, process for obtaining them and applications | 2015-07-24 | UNIVERSIDADE FEDERAL DO PARANA - UFPR |
| 61965465 | BR102015032498 BR102015032498 | (BR102015032498) kit for Immunodiagnostic of Leukemia, Method and Uses | 2015-12-23 | UNIVERSIDADE FEDERAL DE MINAS GERAIS - UFMG |
| 65237424 | BR102017013604 BR102017013604 | (BR102017013604) recombinant Conformational Peptide, Kit and Method for Diagnosing Leciosis, and Use | 2017-06-22 | FUND EDUCACIONAL DE CRICIUMA FUCRI UNIVERSIDADE FEDERAL DE MINAS GERAIS - UFMG |
| 67250518 | BR102017017125 BR102017017125 | (BR102017017125) kit e método molecular para detecção de leishmaniose visceral | 2017-08-09 | UNIVERSIDADE ESTADUAL PAULISTA JULIO DE MESQUITA FILHO - UNESP |
| 67253378 | BR102017022744 BR102017022744 | (BR102017022744) recombinant Antigen Lecithin and Nucleotide Sequence Encoding the Lecithin | 2017-10-23 | UNIVERSIDADE FEDERAL DO PARANA - UFPR |
| 67251542 | Manually harmonized family (**BR102014013193**/BR102015012623) | (BR102015012623) method and kit for diagnosing penicillosis utilizing synthetic peptides derived from the gene encoding mitogen-activated protein kinase 3 (putative) | 2014-05-30 | UNIVERSIDADE FEDERAL DE MINAS GERAIS - UFMG |
| 63451592 | BR102014031331 BR102014031331 | (BR102014031331) recombinant multiepitope protein, its process of obtaining, and its applications related to tuberculosis | 2014-12-15 | UNIVERSITY FEDERAL SÃO JOÃO DEL REI |
| 62558134 62558134 | WOIB2017/058080 BR102016029501 BR102016029501 | (WO2018109753) Conformational peptide, method and kit for diagnosing visceral leishmaniasis | 2016-12-15 | FUND EDUCACIONAL DE CRICIUMA FUCRI UNIVERSIDADE FEDERAL DE MINAS GERAIS - UFMG |
| 63406360 | US20200048722/US10072309 | (US10072309) Methods for real-time multiplex isothermal detection and identification of bacterial, viral, and protozoan nucleic acids | 2015-05-08 |  |
| 63623844 | CN201810272384 | (CN108588252) Parasite detection kit and detection method thereof | 2018-03-29 | HANGZHOU TESTSEA BIOTECHNOLOGY |
| 64268947 | BR102017005135 BR102017005135 | (BR102017005135) method, Kit for Diagnostic of Leciosis and Use | 2017-03-14 | UNIVERSIDADE FEDERAL DE MINAS GERAIS - UFMG |
| 49237533 49237533 49237533 49237533 | EP13766679 EP13766679 US14/902,234 US14/902,234 WOIB2013/055423 BR112015032978 IN201617002973 ES13766679T | (EP3017305) Peptides and methods for the detection of leishmaniasis | 2013-07-02 | IRD - INSTITUT DE RECHERCHE POUR LE DEVELOPPEMENT UNIVERSIDAD PERUANA CAYETANO HEREDIA UNIVERSITY PERUANA CAYETANO HEREDIA |
| 68233396 | ES201830379 | (ES2727968) Method for the diagnosis of cutaneous leishmaniasis | 2018-04-18 | UNIVERSITAT ROVIRA I VIRGILI |
| 71451251 | BR102018003443 BR102018003443 | (BR102018003443) primers para diagnóstico molecular de leishmanioses, kit, método e usos | 2018-02-22 | UNIVERSIDADE FEDERAL DE MINAS GERAIS - UFMG |
| 70547312 | BR102018016009 BR102018016009 | (BR102018016009) recombinant protein, method and kit for diagnosing visceral and integumentary malignancies in man and dog | 2018-08-06 | UNIVERSIDADE FEDERAL DE MINAS GERAIS - UFMG |
|  | BR102018067827 BR102018067827 | (BR102018067827) Recombinant lecidomonas infantum protein, diagnostic kit for visceral albicans, process of detection of visceral albicans, and applications | 2018-09-04 | BRASÍLIA FUNDAÇÃO UNIVERSIDADE |
| 69642685 | WOBR2019/050356 BR102018067339 | (EP3812768) Method for detecting antibodies against leishmania in a canine biological sample, kit for detection of leishmania infection in a biological sample, and use of leishmania lipophosphoglucan | 2018-08-31 | FUNDACAO OSWALDO CRUZ FIOCRUZ |
| 74501993 | Manually harmonized family (BR132017028144/**BR102012032499**) | (BR132017028144) recombinant Protein, Human and Canine visceral psychiatric diagnostics Kit, Medicinal Composition and Uses | 2012-12-19 | FUNDACAO DE AMPARO A PESQUISA DO ESTADO DE MINAS GERAIS FAPEMIG UNIVERSIDADE FEDERAL DE MINAS GERAIS - UFMG |
|  | BR102018067309 | (BR102018067309) Recombinant lpg3 protein of lecidomonas infantiasi, its linear peptides and its uses | 2018-08-31 | GERAIS FAPEMIG FUNDAÇÃO DE AMPARO A PESQUISA DO ESTADO DE MINAS PRETO UNIVERSIDADE FEDERAL DE OURO VIÇOSA UNIVERSIDADE FEDERAL |
| 74042484 | BR102018017162 BR102018017162 | (BR102018017162) recombinant protein, process of production thereof, use of the protein in the preparation of an immunogen against canine or human visceral leukemia, and use of the protein in detection assay or detection kit for detecting asymptotic or subclinical visceral leukemia in a biological sample | 2018-08-22 | EMPRESA GREENBEAN BIOTECNOLOGIA UNIVERSIDADE ESTADUAL DO CEARA UECE |
| 72143290 | WOBR2020/050044 BR102019003639 | (EP3929210) Chimeric protein, method of production and use thereof, and also a nucleic acid molecule, expression cassette, expression vector, host cell, composition for the diagnosis of leishmaniasis, kit for the diagnosis of leishmaniasis and method of diagnosis of leishmaniasis in vitro | 2019-02-22 | FUNDACAO OSWALDO CRUZ FIOCRUZ |
| 74568974 | BR102019005228 | (BR102019005228) primers, kit and method for molecular diagnostics of integumentary leukemia, and use | 2019-03-18 | UNIVERSIDADE FEDERAL DE MINAS GERAIS - UFMG |
|  | BR102019014136 BR102019014136 | (BR102019014136) Bioelectrode for diagnosing lesiasis | 2019-07-08 | UBERLÂNDIA UNIVERSIDADE FEDERAL |
|  | BR102019020805 BR102019020805 | (BR102019020805) conductive polymer nanostructured films, process of obtaining and their use as nucleic acid sensing platforms | 2019-10-03 | UNIVERSIDADE FEDERAL DE PERNAMBUCO - UFPE |
| 69274594 | CN201911301367 CN201911301367 | (CN110734995) Primer pair, probe, detection method and reagent kit for detecting leishmania | 2019-12-17 | BEIJING FRIENDSHIP HOSPITAL CAPITAL MEDICAL UNIVERSITY |
|  | Manually harmonized family **(BR102018073191**/BR102019023354) | (BR102019023354) Recombinant protein, method and kit for diagnostic of viscosis of viscosity and use | 2018-11-09 | UNIVERSIDADE FEDERAL DE MINAS GERAIS - UFMG |
| 69723211 | WOUS2018/049868 | (WO202050852) Methods for real-time multiplex isothermal detection and identification of bacterial, viral, and protozoan nucleic acids | 2018-09-07 |  |
| 76825185 | BR102019026180 | (BR102019026180) kit for diagnosing and/or quantifying the protozoan parasite load: method, oligonucleotides and probes | 2019-12-10 | UNIVERSIDADE FEDERAL DO PARANA - UFPR |
| 72001877 | CN202010382952 | (CN111549160) Primer group and kit for detecting leishmania and application of primer group and kit | 2020-05-08 |  |
|  | BR102020008460 | (BR102020008460) Kit, method for diagnosing visceral leukemia and use | 2020-04-28 | GERAIS UNIVERSIDADE FEDERAL DE MINAS |
| 72258628 | CN202010752650 CN202010752650 | (CN111621583) Method for population typing and gene tracing of Leishmania | 2020-07-30 | BEIJING FRIENDSHIP HOSPITAL CAPITAL MEDICAL UNIVERSITY |
|  | BR102020007615 | (BR102020007615) Psli379 synthetic peptide and canine visceral leukemia immunodiagnostic kit | 2020-04-16 | PARANÁ UNIVERSIDADE FEDERAL |
| 73201387 | CN202010745529 | (CN111876512) Reagent and kit for detecting two leishmania by isothermal amplification and application of reagent and kit | 2020-07-29 | SHENZHEN CENTER FOR DISEASE CONTROL & PREVENTION |
|  | BR102020009366 | (BR102020009366) Elisa kit and method for immunodiagnostic of human visceral and cutaneous and canine visceral leukemia utilizing novel synthetic peptide antigen psli409 | 2020-05-12 | PARANÁ UNIVERSIDADE FEDERAL |
| 75816770 | CN202110387134 | (CN112795676B) Kit for identifying visceral leishmaniasis infection pathogen insect species | 2021-04-12 | JIANGSU BIOPERFECTUS TECHNOLOGY |
| 75816912 | CN202110392040 | (CN112795677B) Kit for identifying skin leishmania species | 2021-04-13 | JIANGSU BIOPERFECTUS TECHNOLOGY |
| 78791904 | CN202111004281 | (CN113755620) Nucleic acid detection kit for rapidly detecting two leishmania and application thereof | 2021-08-30 | JIANGSU PROVINCE **BLOOD** CENTER |
| 79194350 | CN202111341030 | (CN113897448) Primer, method and kit for performing multiplex tandem type PCR (polymerase chain reaction) gene disc detection on Leishmania | 2021-11-12 | SICHUAN INTERNATIONAL TRAVEL HEALTH CARE CENTER CHENGDU CUSTOMS PORT OUTPATIENT DEPARTMENT |
| 81779311 | CN114574607 | (CN114574607) Kit and application thereof | 2022-05-05 | BEIJING FRIENDSHIP HOSPITAL CAPITAL MEDICAL UNIVERSITY |
| 82446318 | BR102021000794 | (WO2022150899) Proteína quimérica, kit, método para diagnóstico de leishmaniose, uso de uma proteína quimérica, composição vacinal contra leishmaniose visceral, e, uso de uma composição vacinal | 2021-01-15 | FUNDACAO OSWALDO CRUZ FIOCRUZ |
|  | BR102020025878 | (BR102020025878) Kit, método para o sorodiagnóstico de leishmaniose visceral e uso | 2020-12-17 | GERAIS UNIVERSIDADE FEDERAL DE MINAS HORIZONTE SANTA CASA DE MISERICÓRDIA DE BELO |
| 81753628 81753628 | BR102020023981 | (WO2022109690) Método de diagnóstico em uma amostra biológica, kit de diagnóstico em uma amostra biológica e oligonucleotídeo | 2020-11-24 | FUNDACAO OSWALDO CRUZ FIOCRUZ |
| 80855927 | BR102020015591 | (BR102020015591) Proteína quimérica recombinante, kit e método para diagnóstico de leishmaniose tegumentar e usos | 2020-07-30 | UNIVERSIDADE FEDERAL DE MINAS GERAIS - UFMG |
|  | BR102019004212 | (BR102019004212) Proteína quimérica recombinante multiepitopo e suas aplicações para leishmanioses | 2019-02-28 | REI UNIVERSIDADE FEDERAL DE SÃO JOÃO DEL |
