# Supplementary material for: Developments in Leishmaniasis diagnosis: A patent landscape from 2010 to 2022
Source: PLOS Glob Public Health. 2023 Nov 1;3(11):e0002557. doi: 10.1371/journal.pgph.0002557 (PMC10619796; doi:10.1371/journal.pgph.0002557)
Supplement: S1 Text — (DOCX) [file pgph.0002557.s002.docx]

**S1 Text- Search strings**

1. (LEISHMANIA+)/TI/AB/CLMS AND (G01N 33/569 OR C12Q 1+ OR G01N+)/IPC/CPC AND (EPRD >= 2010-01-01)
2. (DIAGNOS+)/TI/AB/CLMS AND (A61K-039/008)/IPC/CPC AND (EPRD >= 2010-01-01)
3. (LEISHMANI+ AND +DIAGNOS+)/TI/AB AND (EPRD >= 2010-01-01)
4. (LEISHMANI+ AND +DIAGNOS+)/CLMS AND (EPRD >= 2010-01-01)
5. (LEISHMANI+ AND DETECT+)/TI/AB/CLMS AND (EPRD >= 2010-01-01)
6. (LEISHMANIA)/CLMS AND (C07K-014/44)/IPC/CPC AND (EPRD >= 2010-01-01)
7. 1 OR 2 OR 3 OR 4 OR 5 OR 6
