# Supplementary material for: Developments in Leishmaniasis diagnosis: A patent landscape from 2010 to 2022
Source: PLOS Glob Public Health. 2023 Nov 1;3(11):e0002557. doi: 10.1371/journal.pgph.0002557 (PMC10619796; doi:10.1371/journal.pgph.0002557)
Supplement: S2 Text — (DOCX) [file pgph.0002557.s003.docx]

**S2 Text - Manually grouped patent families**

1. US2016130669/WO201506755; (ii) BR132017028144/BR102012032499; (iii) BR102014013195/BR102015012622; (iv) BR102014013193/BR102015012623; (v) BR102012032022/BR102013031983/WO201491463; (vi) BR102018073191 /BR102019023354; (vii) BR201105461/BR132013001271/WO201219268
