# Supplementary material for: Developments in Leishmaniasis diagnosis: A patent landscape from 2010 to 2022
Source: PLOS Glob Public Health. 2023 Nov 1;3(11):e0002557. doi: 10.1371/journal.pgph.0002557 (PMC10619796; doi:10.1371/journal.pgph.0002557)
Supplement: S3 Text — (DOCX) [file pgph.0002557.s004.docx]

**S3 Text - Full protein description**

Several 1 - hypothetical proteins, Putative Fatty acid Elongase, Peroxidoxin 1, Heat shock protein 83-1 beta-tubulin, HSP-83-1, HSP83, HSP90,sterol-24c-methyltransferase, tryparedoxin peroxidase, 14-3-3 protein like protein, IgE dependent histamine releasing factor, Prostaglandin f2-alpha synthase, putative eukaryotic initiation factor 4a, aldose 1-epimerase, mannose-1-phosphate guanyltransferase, alpha tubulin, phosphomannomutase, HSP 70, actin, eukaryotic translation initiation factor 3 subunit, adenosine kinase, putative ribonucleoprotein p18 mitochondrial precursor, s-adenosylhomocysteine hydrolase, mitochondrial precursor of 3,2-trans-enoyl-CoA isomerase, pyruvate dehydrogenase E1 beta subunit, elongation factor 1 beta, nascent polypeptide associated to homologue complex, subunit of eukaryotic initiation factor 3.

Several 2 - Putative trypanothione synthase, metalo-peptidase Clan MA(E) protein, enolase, Elongation factor 2, hypothetic proteins, peptidase m20/m25/m40 , putative heat shock protein, putative aconitase, putative beta chain of succinyl-CoA ligase, putative mitochondrial peroxiredoxin, putative IgE dependant histamin liberation factor, paraflagelar rod 1, myristoylated protein, zeta-cristalin/NADPH-oxiredutase-like protein, putative HSP-70

Several 3 - CAS/CSE/importin domain protein, endonuclease III, paraflagellar rod 1C and 1D, elongation factor 2, pyridoxal kinase, GTP-binding protein, polynicleotide adenyltransferase, elongation factor 1beta, hypothetical proteins

Several 4 - Uncharacterized proteins, DNA-directred RNA polymerases, 1-acyl-sn-glycerol-3-phosphateacyltransferase-like-prtein, Histone H2A, Histone H2A1, putative protein kinase, hydrophilic acylated surface protein, k26, aquaglycerolporin, putative ATPase domain protein, putative mitochondrial chaperone BCS1, ENOL protein, putative inositol polyphosphate phosphatase, hydrophilic acylated surface protein, putative kinesin, RNAse III domain containing protein, Histone H2B, putative 2-oxoglutarate dehydrogenase subunit, DNA-directed RNA polymerase subunit beta.
