# Supplementary material for: Developments in Leishmaniasis diagnosis: A patent landscape from 2010 to 2022
Source: PLOS Glob Public Health. 2023 Nov 1;3(11):e0002557. doi: 10.1371/journal.pgph.0002557 (PMC10619796; doi:10.1371/journal.pgph.0002557)
Supplement: Attachment — Submitted filename: Rebuttal letter.docx [file pgph.0002557.s005.docx]

Dear Marianne,

First, we would like to thank you and both reviewers for your considerations. We have addressed all the points raised in the reviewing process and made the necessary amendments. Changes are tracked in the word document.

Regarding Journal Requirements mentioned in your email, we have (i) added the full list of legends for our Supporting Information files after the references list (modifications were made on the title of each supporting information file to avoid redundancy with the legends – a new file named supporting-information-revised-manuscript is therefore included in this submission) (ii) reviewed the reference list to ensure that it is complete and correct and (iii) sent pictures to PACE.

Our reply to reviewers’ comments is given below:

**Reviewer #1**

**1. Line 83: “results” should replace “resulting”**

Correction made.
**2. Check spacing consistency between words and sentences.**

Spacing consistency checked throughout the text.
**3. Line 412: rK39, should K be capitalized? Small case is used throughout, k39**

Yes. It should be. Change made.
**4. Line 481: needs a period after the citation bracket.**

Correction made.

**Reviewer #2:**

**1. Review the text for typos or missing words,**

A through revision of the text was made, and corrections were made for typos, missing words, grammatical errors. Changes for the sake of text clarity where also made (paragraphs 402 and 428).

**examples:
1a. Page 1 Abstract "...revealed are consistent WITH the needs..."**

Correction made
**1b. Page 8, line 86. "...published IN the scientific litearature..."**

Correction made
**1c. Page 8, line 93. "...consistent WITH the needs..."**
Correction made
**2. Page 7, line 78. PLease note that the target product profile mentioned is for dermal leishmaniasis, not only for cutaneous leishmaniasis (CL).**

Correction made

**3. It may worth mentioning the development of target product profiles for diagnostic tests for visceral leishmaniasis** [**https://www.who.int/news-room/articles-detail/call-for-public-consultation-----target-product-profiles-(tpp)-for-visceral-leishmaniasis-diagnostics**](https://www.who.int/news-room/articles-detail/call-for-public-consultation-----target-product-profiles-(tpp)-for-visceral-leishmaniasis-diagnostics)

Thank you for bringing this important document to our attention. We have added a paragraph to describe the DTAG-NTD (see lines 58-62) and their draft target public profiles for visceral leishmaniasis (lines 75-82). References were added (see refence 5, 6 and 8).

**4. It may worth mentioning work done by the WHO Diagnostic Technical Advisory Group for NTDs https://www.who.int/groups/diagnostic-technical-advisory-group-for-neglected-tropical-diseases/about-us#:~:text=WHO%20has%20established%20a%20Diagnostic,process%20is%20able%20to%20properly
And the report of their different meetings, where priorities for leishmaniasis diagnostic tests are discussed, example: https://www.who.int/publications/i/item/9789240003590
Reports on up to the fifth consultative meeting can be found in who web site.**

We added to the introduction a paragraph about DTAG-NTD, as indicated in the previous topic. We also added to the discussion information regarding the need for development of a multiplex diagnostic platform for Skin NTDs, topic under evaluation by DTAGs subgroups and discussed at their meetings (Lines 495-496). References were added (40 and 41).
