# Supplementary material for: Developments in Leishmaniasis diagnosis: A patent landscape from 2010 to 2022
Source: PLOS Glob Public Health. 2023 Nov 1;3(11):e0002557. doi: 10.1371/journal.pgph.0002557 (PMC10619796; doi:10.1371/journal.pgph.0002557)
Supplement: Attachment — Submitted filename: responsetoreviewers-out23.docx [file pgph.0002557.s006.docx]

Dear Suma Thankamma Krishnasastry,

Thank you for the careful considerations. We noted that the points presented had already been addressed in our last revision. It seems we made a mistake on the upload of our revised versions on the editorial manager. While the version with tracked changes was the corrected version, the clean version (named “manuscript”), was in fact the original version. I am very sorry for this inconvenience. We have addressed again all points specified:

1. Review the text for typos / missing words/ spacing consistency  etc.

Another revision done and corrections were made.

2. rk39 test should be corrected to  rK39 and maintain consistency in all instances

We verified that capitalized “K” was used in all instances.

3. Page 7, line 78. Please note that the target product profile mentioned is for post kala azar dermal Leishmaniasis (PKDL) and also for cutaneous leishmaniasis (CL)

We have changed the wording to: “A 2019 TPP for a point of care test for dermal leishmaniasis considers the following specific optimal features: species-specific detection of any form of CL or PKDL with more than 95% specificity”. The “dermal leishmaniasis” is the terminology used in the TPP.

4. It may be worth mentioning work done by the WHO Diagnostic Technical Advisory Group for NTDs <https://www.who.int/groups/diagnostic-technical-advisory-group-for-neglected-tropical-diseases/about->

We have included mentions to WHO DTAG.

Page 3 line 60

“Considering diagnostics directly inform several targets set in the 2021-2030 roadmap, WHO established in 2019 a Diagnostic Technical Advisory Group for NTDs (DTAG-NTD) to address priority areas for NTD diagnostics, identify gaps in access, and advise on developments required for the diagnostic process to properly inform decisions on NTD treatment”

Page 4 Line 75

“Target Product Profiles (TPPs) for point of care *in vitro* detection of active VL and for *in vitro* laboratory-based test of cure for VL post-treatment have recently undergone public consultation. Draft versions of the TPPs highlight the need of diagnostic tests with the following ideal features: detection of analytes specific to *L. donovani* or *L. infantum,* with more than 95% clinical sensitivity and 99% clinical specificity; qualitative result for detection of active VL and quantitative result to confirm VL cure; execution under zero-infrastructure conditions, at low cost, using peripheral whole blood from finger stick, urine or saliva as samples, and enabling test result within 30 minutes”

Page 35 Line 498

“In fact, the need for a multiplex platform for skin NTD diagnosis is on DTAG’s agenda, the requirements of which are still to be defined”

Best regards,

Alice Faioli
